# Overexpression of the First Peanut-Susceptible Gene, *AhS5H1* or *AhS5H2*, Enhanced Susceptibility to *Pst* DC3000 in Arabidopsis

**DOI:** 10.3390/ijms241814210

**Published:** 2023-09-18

**Authors:** Bingbing Liang, Yuanjun Bai, Chaoqun Zang, Xue Pei, Jinhui Xie, Ying Lin, Xiaozhou Liu, Taswar Ahsan, Chunhao Liang

**Affiliations:** 1 Institute of Plant Protection, Liaoning Academy of Agricultural Sciences, Shenyang 110161, China; liangbb2121@163.com (B.L.);; 2 Institute of Rice Research, Liaoning Academy of Agricultural Sciences, Shenyang 110101, China

**Keywords:** salicylic acid, susceptible genes, salicylate hydroxylase, *Arachis hypogaea*, Arabidopsis

## Abstract

Salicylic acid (SA) serves as a pivotal plant hormone involved in regulating plant defense mechanisms against biotic stresses, but the extent of its biological significance in relation to peanut resistance is currently lacking. This study elucidated the involvement of salicylic acid (SA) in conferring broad-spectrum disease resistance in peanuts through the experimental approach of inoculating SA-treated leaves. In several other plants, the salicylate hydroxylase genes are the typical susceptible genes (*S* genes). Here, we characterized two SA hydroxylase genes (*AhS5H1* and *AhS5H2*) as the first *S* genes in peanut. Recombinant AhS5H proteins catalyzed SA in vitro, and showed SA 5-ydroxylase (S5H) activity. Overexpression of *AhS5H1* or *AhS5H2* decreased SA content and increased 2,5-DHBA levels in Arabidopsis, suggesting that both enzymes had a similar role in planta. Moreover, overexpression of each *AhS5H* gene increased susceptibility to *Pst* DC3000. Analysis of the transcript levels of defense-related genes indicated that the expression of *AhS5H* genes, *AhNPR1* and *AhPR10* was simultaneously induced by chitin. Overexpression of each *AhS5H* in Arabidopsis abolished the induction of *AtPR1* or *AtPR2* upon chitin treatment. Eventually, *AhS5H2* expression levels were highly correlated with SA content in different tissues of peanut. Hence, the expression of *AhS5H1* and *AhS5H2* was tissue-specific.

## 1. Introduction

The cultivated peanut (*Arachis hypogaea* L.) is a highly popular and widely consumed oil crop across the globe. It is cultivated in over 100 countries [1]. However, there are numerous types of pathogens that pose a significant threat to peanut cultivation and overall crop yield. The control of pathogens in peanut cultivation often involves the use of large quantities of fungicides, which can pose significant risks to human health and the environment. 

Salicylic acid (SA), also known as 2-hydroxybenzoic acid, is a plant hormone. It serves as a mediator for plant defense responses against both biotic and abiotic stresses. In addition, SA plays a crucial role in regulating various physiological and biochemical processes in plants [2,3]. SA can be generated in plants via two enzymatic pathways, that is, isochorismate (ICS), and phenylalanine ammonia lyase (PAL)-mediated pathways, which require the same primary metabolite chorismate [2]. Studies on mutants deficient in SA biosynthesis or signaling revealed that SA is essential to PTI and ETI in local tissue and systemic acquired resistance (SAR) over long distances [4,5,6].

An increase in SA levels activates the transcription of pathogenesis-related (*PR*) genes in Arabidopsis [6,7]. Plants accumulating low SA levels show decreased oxidative stress and increased shoot and seed number, suggesting that SA has a negative role in plant growth and development [3,8,9,10].

In peanut, the root endophyte *Phomopsis liquidambaris* B3 effectively protected peanut against *Fusarium oxysporum* by activating defense, which positively correlated with SA [11]. The biological importance of SA in peanut resistance remains deficient.

Although the dominant resistance (*R*) genes have been used to develop resistant varieties through the ages, resistance mediated by a single *R* gene frequently lacks durability due to the loss or mutation of the *R* gene’s cognate molecule (effector) of pathogens [12]. The strategy of combining multiple *R* genes in one genotype, also known as “gene stacking”, is a more effective approach for maintaining crop resistance [13,14]. Another strategy for acquiring resistant plants is to inactivate the so-called susceptibility genes (*S*), which promote infection and mediate compatible interactions with pathogens [15]. One well-known *S* gene is *Mildew Locus O* (*MLO*), whose mutations are linked to resistance to powdery mildew in many crops [16,17,18,19,20,21,22]. 

No *S* genes were known to confer resistance to pathogens in peanuts, but *S* genes are usually conserved among plant species [23]. In Arabidopsis, the *downy mildew resistant* (*dmr6*) mutant has increased resistance against the oomycete pathogen *Hyaloperonospora parasitica*, and DMR6 was cloned as a member of 2-oxoglutarate-dependent dioxygenases (2OGDs) [24]. DMR6 and its close homolog DLO1 (DMR6-like oxygenase1) act as partially redundant, but distinct, suppressors of immunity [25]. Later, DMR6 and DLO1/S3H1 were identified as SA 5-hydroxylase (S5H) and 3-hydroxylase (S3H), respectively [26,27]. In the *dmr6 dlo1*/*s3h1* double mutant, the SA level is further elevated compared with that of the *dmr6* single mutant, leading to immunity to pathogens and strong growth retardation [25,27]. In the last few years, orthologs of the *DMR6* and *DLO* genes have been identified and proved to be *S* genes in several crops such as tomato, banana, grapevine and rice [28,29,30,31].

This paper demonstrates the significant role of salicylic acid in enhancing peanuts’ broad-spectrum disease resistance through the inoculation of leaves treated with SA. We subsequently characterized the primary SA hydroxylases, AhS5H1 and AhS5H2, from peanut. The function of salicylate hydroxylases were confirmed both in vitro and in transgenic Arabidopsis plants. Furthermore, the significance of salicylate hydroxylase genes in the defense response was confirmed through the detection of defense-related gene expression by applying chitin treatment. The biochemical functions of AhS5H1 and AhS5H2 were highly similar. However, the expression of *AhS5H1* and *AhS5H2* in peanuts was specific to certain tissues.

## 2. Results

### 2.1. Peanut Resistance to Multiple Pathogens Was Induced by Exogenous SA Treatment

Although SA is a plant immune signal essential for many species of plants, the role played in peanut resistance to pathogens remained unclear. Therefore, we inoculated peanut (Baisha1016) with multiple common peanut pathogens after treatment with 1 mM salicylic acid at 24 hpt (hours post treatment). Plants inoculated without treatment were used as controls.

**Figure 1:**
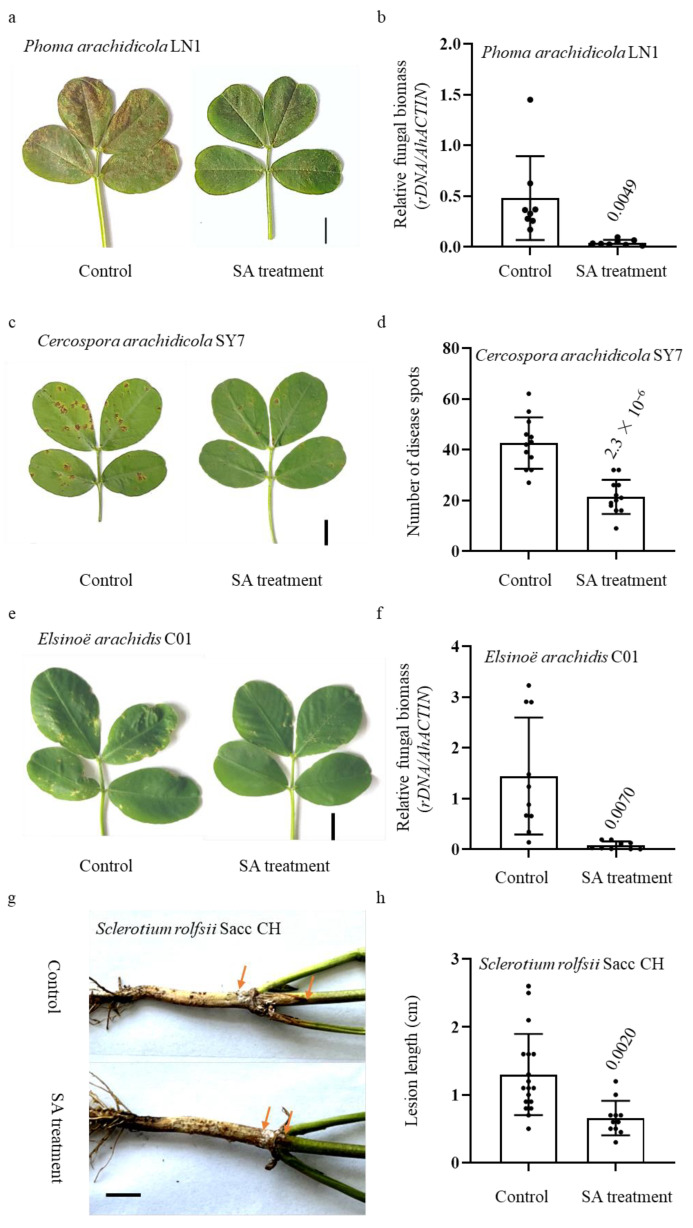
Peanut resistance to multiple pathogens was induced by exogenous SA treatment. Disease symptoms and severity statistics are shown. Disease phenotypes (**a**) and severity of *P. arachidicola* (**b**). Three-week-old plants were inoculated with *P. arachidicola* LN1 isolate (2 × 10^6^ conidia/mL) by foliar spraying; evaluation of disease severity and photography taken were conducted at 7 d post inoculation. The relative fungal biomass was evaluated by qRT-PCR using *P. arachidicola* 26S rDNA and peanut *AhACTIN* gene (*n* = 8). Disease phenotypes (**c**) and severity of *C. arachidicola* SY7 (**d**). Three-week-old plants were inoculated with *C. arachidicola* SY7 isolate (5 × 10^5^ conidia/mL) by foliar spraying; evaluation of disease severity and photography taken were conducted at 8 d post inoculation. Disease numbers were determined (*n* = 12). Disease phenotypes (**e**) and severity of *E. arachidis* (**f**). One-month-old plants were inoculated with *E. arachidis*. Evaluation of disease severity and photography taken were conducted at 10 d post inoculation. The relative fungal biomass was evaluated by qRT-PCR using *E. arachidis*. 26S rDNA and peanut *AhACTIN* gene (*n* = 10). Disease phenotypes (**g**) and lesion lengths of *Sclerotium rolfsii* Sacc CH (**h**). Four-week-old plants were inoculated with *S. rolfsii* Sacc CH isolate. A slice of filter paper containing the *S. rolfsii* Sacc CH was pinned around the bottom of the stem. Lesion lengths were measured 6 d after the inoculation (*n* = 15). *p* value evaluated using Student’s *t*-test is above the boxplot. Bar = 1 cm.

In comparison to the control samples, it was observed that plants subjected to SA treatment exhibited significantly reduced disease lesions upon exposure to *Phoma arachidicola* LN1, a highly virulent strain of the peanut web blotch fungus (Figure 1a). Furthermore, it is noteworthy to mention that the fungal biomass observed within the leaves exhibited a decrease when compared to the control group, as depicted in Figure 1b. Early leaf spot resistance was assessed through the application of spores via foliar spraying on plants that were three weeks old. The control peanuts exhibited a higher susceptibility to the *Cercospora arachidicola* SY7 isolate, while leaves treated with SA displayed a reduced number of diseased spots (Figure 1c,d). In a similar vein, the plants underwent inoculation with *Elsinoë arachidis* C01, a pathogenic agent known for inducing peanut scab. The observed reduction in the number of disease spots on the leaves of plants treated with SA was found to be statistically significant compared to the control plants. This decrease in disease spots was also accompanied by a decrease in fungal biomass, as depicted in Figure 1e,f.

It is often reported that SA promotes resistance against both local defense response and SAR [32]. Hence, the basal stem of SA-treated and control plants was inoculated with *S. rolfsii* Sacc CH, a virulent peanut stem rot strain, to investigate its important role in peanut SAR. We observed that the lesion lengths of SA-treated plants were significantly shorter than the control ones (Figure 1g,h). These results collectively indicate that peanut resistance to multiple pathogens was induced by exogenous SA treatment.

### 2.2. Phylogenetic Analysis and Characterization of SA Hydroxylases in Peanut

In several other species of plant, orthologs of *DMR6* or *DLO1* regulate SA homeostasis and broad-spectrum resistance to pathogens [27,28,29,30,31]. Therefore, we took into account the necessity of screening Arabidopsis S3H or S5H homologs in peanut.

**Figure 2:**
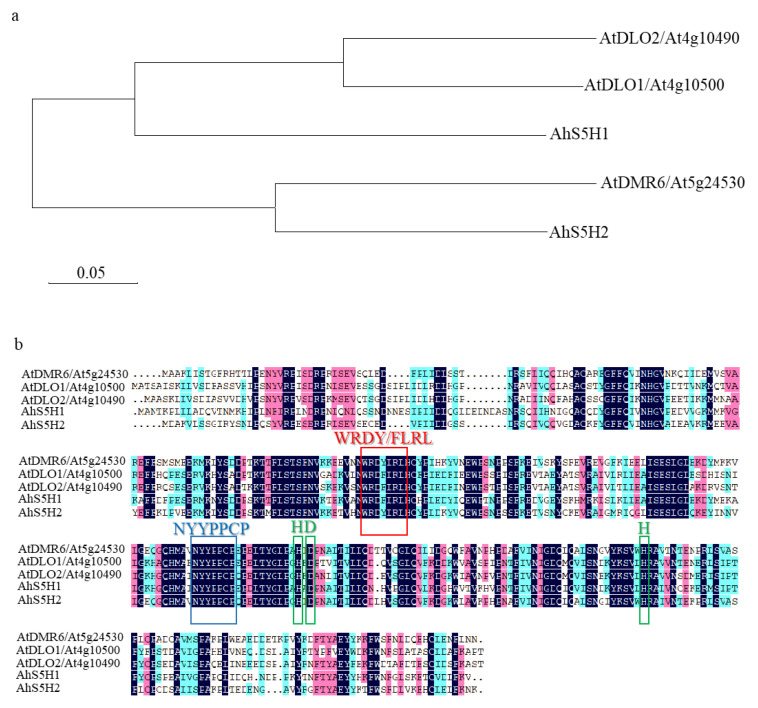
Phylogenetic tree and sequence alignment. (**a**) The alignment was colored with the default DNAMAN color scheme according to the amino acid chemical properties. The ordering of the sequences was based on pairwise similarity. Motifs that were important for the catalytic function were marked by colored boxes: the HDH motif (green), the NYYPPCP motif (blue), and the WRDY/FLRL motif (red)—specific to the DMR6 and DLO proteins. (**b**) The evolutionary history was inferred using the Neighbor-Joining method. The optimal tree with the sum of branch length = 1.15622734 was shown. The tree was drawn to scale, with branch lengths in the same units as those of the evolutionary distances used to infer the phylogenetic tree. The evolutionary distances were computed using the Poisson correction method and were in the units of the number of amino acid substitutions per site. Evolutionary analyses were conducted in MEGA7.

Based on conserved domains of the conserved 2-oxoglutarate-dependent dioxygenases, we used the protein sequences of Arabidopsis S3H and S5H to blast the NCBI database (https://www.ncbi.nlm.nih.gov/ accessed on 22 January 2022). As shown in Figure 2a, two homologs were identified in peanut and named AhS5H1 (XP_025631840), AhS5H2 (XP_025683565). AhS5H1 displayed high similarity to Arabidopsis S3H and its close homolog DLO2 [25,26] due to the phylogenetic analysis, and AhS5H2 was similar to S5H [27].

The open reading frame (ORF) of AhS5H1 and AhS5H2 was validated to be 1059 bp and 1014 bp. Respectively, the amino acid sequences of AhS5H1 and AhS5H2 were 353 and 338 in length. 

Figure 2b illustrates the identification of conserved patterns observed in both (2OG)-Fe(II) oxygenases and the DMR6-DLO clade. These patterns are represented by colored boxes and signify common motifs and specific residues. The HDH motif is responsible for binding the catalytic iron (Fe II), whereas the NYYPPCP motif plays a crucial role in interacting with the 2-oxoglutarate substrate. The WRDY/FLRL motif has been postulated to play a role in the binding of SA [33]. As a result, AhS5H1 and AhS5H2 were chosen as candidate SA hydroxylases for further study.

### 2.3. Identification of the Salicylic Acid 5-Hydroxylases In Vitro

**Figure 3:**
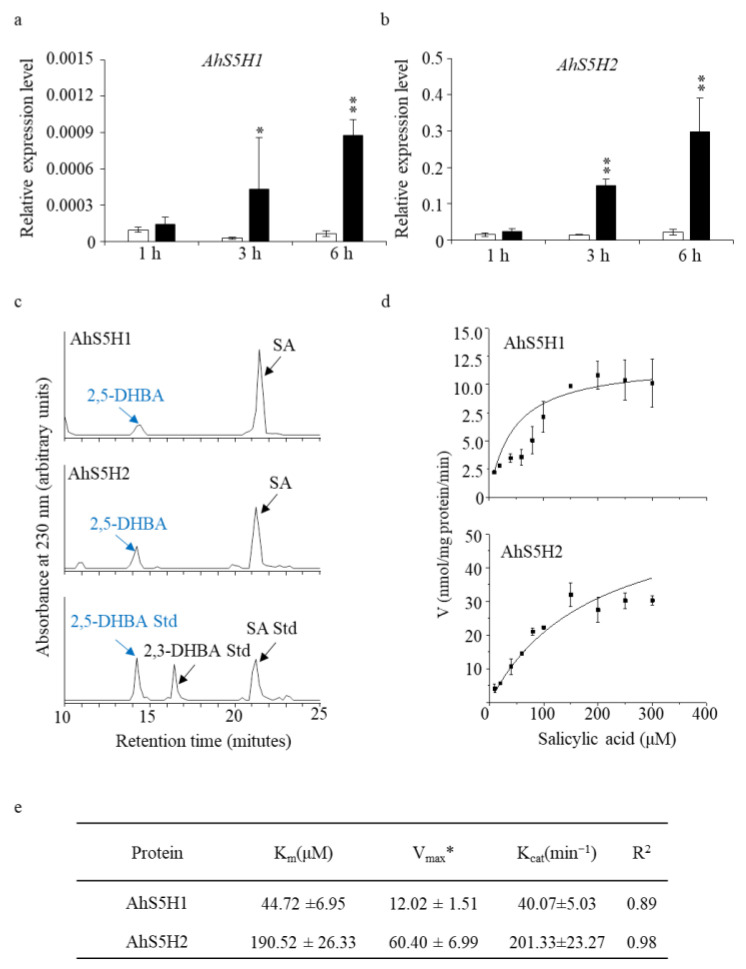
Identification of the salicylic acid 5-hydroxylases in vitro. Expression levels of *AhS5H1* and AhS5H2 in peanuts after SA treatment (**a**,**b**). Three-week-old seedlings were treated with 1 mM SA in 5 mM MES buffer for 1 h, 3 h and 6 h. For the mock treatment, the seedlings received the same volume of DMSO solvent. Gene expression was determined by qRT-PCR using *AhACTIN* as the reference gene. Values are means ± SD (*n* = 3). Asterisks indicate statistically significant differences compared with the corresponding mock using Student’s *t*-test (*, *p* < 0.05; **, *p* < 0.01). (**c**) HPLC profiles of the 30 min reaction of the recombinant AhS5H1 and AhS5H2 proteins on the SA substrate. Authentic 2,5-dihydroxyl benzoic acid (2,5-DHBA) was used as a standard. Kinetics curves (**d**) and kinetic parameters (**e**). * nmol/mg protein/min. *K_m_* for Michaelis constant, *V_max_* for maximum reaction rates, *K_cat_* for catalytic rate constant. Kinetic parameters and curves were obtained from the reactions at pH 6.8 and 40 °C for 30 min. The data are presented as means ± SD (*n* = 3).

To test our hypothesis, we treated peanut seedlings (Baisha1016) with 1 mM SA. As was shown in Figure 3a,b, the expression of *AhS5H1* and *AhS5H2* was strongly induced at the time period tested in contrast to the control seedlings. 

In order to elucidate the biochemical role of the two anticipated SA hydroxylases in an in vitro setting, the recombinant proteins of AhS5H1 and AhS5H2 were isolated from *Escherichia coli* and subjected to enzymatic activity assays. The enzymatic assays revealed that both of the candidate SA hydroxylases demonstrated exclusive SA 5-hydroxylase activities (Figure 3c,d). Figure 3e presents the biochemical parameters pertaining to the AhS5H proteins. The observed maximum reaction rates (*V_max_*) for AhS5H1 and AhS5H2 enzymes on substrate SA were determined to be 12.02 ± 1.51 and 60.40 ± 6.99 nmol/mg protein/min, respectively. The *K_m_* values of AhS5H1 and AhS5H2 were determined to be 44.72 ± 6.95 and 190.52 ± 26.33 µM, respectively. AhS5H2 exhibited a higher level of enzymatic activity, indicating a greater degree of robustness in its enzyme function. Based on our collective analysis, it can be deduced that AhS5H1 and AhS5H2 serve as the primary functional SA 5-hydroxylase enzymes in the peanut species.

### 2.4. Overexpression of the Candidate Peanut Salicylate Hydroxylase Genes Reduced Salicylic Acid Level in Arabidopsis

The full-length *AhS5H1* and *AhS5H2* coding sequences were cloned and placed under the Cauliflower mosaic virus 35S promoter. The obtained constructs (35S::AhS5H1-GFP and 35S::AhS5H2-GFP) and control (35S::GFP) were transformed into *A. tumefaciens* strain GV3101 to generate transgenic Arabidopsis. Two lines of Arabidopsis for each of the two genes were obtained.

**Figure 4:**
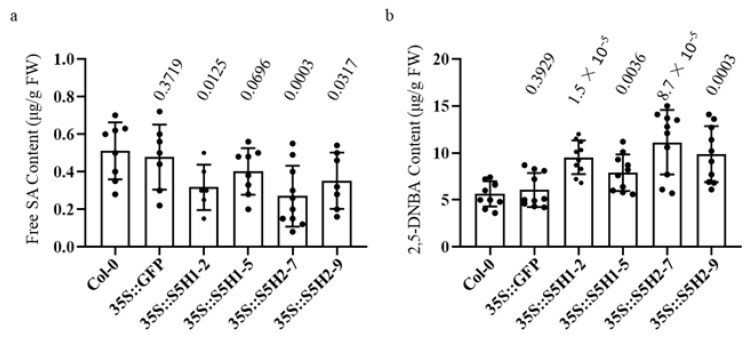
Phenolic accumulation of transgenic Arabidopsis plants. Accumulation of SA (**a**) and 2,5-DHBA (**b**). The amounts of compounds were determined by HPLC. Values are given as means ± SD (*n* ≥ 10). p value evaluated using Student’s *t*-test is above the boxplot. Prefix 35S for overexpressing gene.

The expression of *AhS5H1* and *AhS5H2* was observed to be significantly enhanced in their respective lines that were subjected to overexpression, as depicted in Figure S1. The levels of SA, serving as the substrate for AhS5H1 and AhS5H2, exhibited a significant decrease in comparison to control and wild-type (Col-0) plants at 40 DAG (days after germination) (Figure 4a). Simultaneously, there was a significant enhancement in the production of 2,5-dihydroxybenzoic acid (2,5-DHBA) (Figure 4b). The results indicated that both AhS5H1 and AhS5H2 exhibit hydroxylase activity in plants.

### 2.5. Overexpression of the Candidate Peanut Salicylate Hydroxylase Genes Enhanced Susceptibility to Pst DC3000 in Arabidopsis

**Figure 5:**
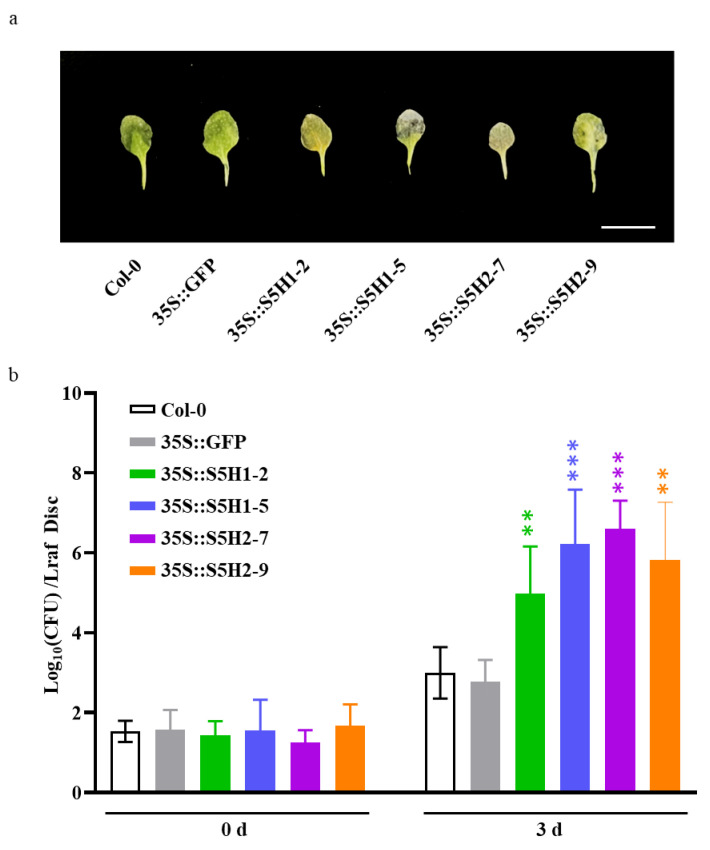
Disease phenotypes and severity of *Pst* DC3000. (**a**) Disease symptoms of three-week-old Arabidopsis plants from the WT, 35S::GFP and *AhS5H* overexpression lines 3 days after *Pst* DC3000 suspension (OD_600_ = 0.05) infiltration. Bar = 1 cm (**b**) The *AhS5H* overexpression lines in the wild-type background were more susceptible to *Pst* DC3000 than the wild-type or 35S::GFP line. CFU, Colony forming units. The data are presented as means ± SD (*n* = 8). **, *p* < 0.01 and ***, *p* < 0.001 (Student’s *t*-test).

We also assessed the pathogen resistance of WT, control (35S::GFP), *AhS5H1* overexpression lines (35S::S5H1-2 and 35S::S5H1-5) and AhS5H2 overexpression lines (35S::S5H2-7 and 35S::S5H2-9). The transgenic and wild-type plants were inoculated with *Pst* DC3000, a common bacterial pathogen, by foliar spraying of a bacterial suspension on three-week-old plants. All the *AhS5H1* and *AhS5H2* overexpression lines displayed enhanced susceptibility to *Pst* DC3000 compared to the WT or control lines. (Figure 5a,b), which is consistent with the phenotypes of the *DMR6* overexpression lines [25,27].

### 2.6. The Expression of AhS5H1 and AhS5H2 Was Defense Associated

**Figure 6:**
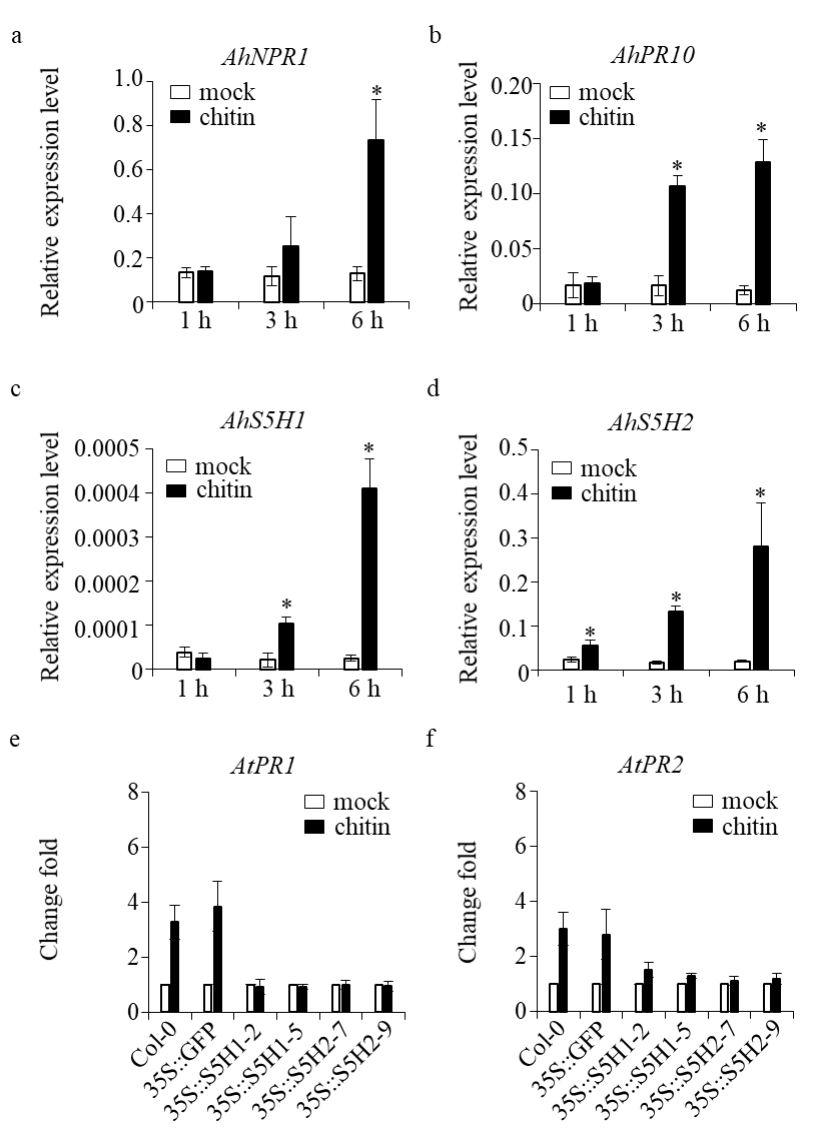
The expression of *AhS5H1* and *AhS5H2* was defense-associated. Induction of defense-related genes (**a**,**b**) and *AhS5Hs* (**c**,**d**) by chitin. Three-week-old peanut plants grown in soil were treated with 100 μg/mL chitin and the same volume of DMSO solvent for the mock treatment. Transcript level was determined by qRT-PCR using *AhACTIN* as the reference gene. Values are given as means ± SD (*n* = 3). Asterisks indicate statistically significant differences compared with the mock (Student’s *t*-test, *, *p* < 0.05). Average fold change in *AtPR1* (**e**) or *AtPR2* (**f**) expression in WT, 35S::GFP and *AhS5H* overexpression lines of Arabidopsis. Three-week-old seedlings was treated with 100 μg/mL chitin. Gene expression in mock samples was set as 1 and the samples treated with chitin were compared with their own mock samples, unless otherwise indicated. Values are given as means ± SD (*n* = 6).

To elucidate the potential alterations in the transcription of SA 5-hydroxylase genes during the defense response, we employed quantitative real-time polymerase chain reaction (qRT-PCR) to determine the relative transcript levels in both peanut and transgenic Arabidopsis. *AhNPR1* and *AhPR10* are genes that play a role in the defense mechanisms of peanut plants, specifically in the salicylic acid (SA) signaling pathway [11]. The expression levels of *AhNPR1* and *AhPR10* were observed to exhibit a statistically significant increase subsequent to chitin treatment, as depicted in Figure 6a,b. In the interim, the transcriptional activity of the *AhS5H1* and *AhS5H2* genes exhibited an up-regulated pattern during the corresponding time period, as shown in (Figure 6c,d).

To gain more information, a set of defense-related genes, such as *AtPR1* and *AtPR2* [34], were subjected to qRT-PCR analysis using six biological replicates for validation. Expression levels were quantified in the wild-type (WT) samples, control (35S::GFP) samples, as well as the overexpression lines of *AhS5H1* (35S::S5H1-2 and 35S::S5H1-5) and *AhS5H2* (35S::S5H2-7 and 35S::S5H2-9). Both tested genes exhibited clear induction in response to chitin stimulation in both wild-type (WT) and control plant samples, when compared to the overexpression lines (Figure 6e,f). The integrated dataset revealed that the expression of *AhS5H1* and *AhS5H2* genes exhibited alterations in the response to defense mechanisms.

### 2.7. The Expression of AhS5H1 and AhS5H2 in Peanut Was Tissue-Specific

As indicated by the results above. The AhS5H1 and AhS5H2 were highly similar in biochemical function and response to chitin treatment. Therefore, in order to find the differences between the two genes, we collected the roots, stems, leaves and flowers of peanut at the flowering stage for the detection of transcript levels.

**Figure 7:**
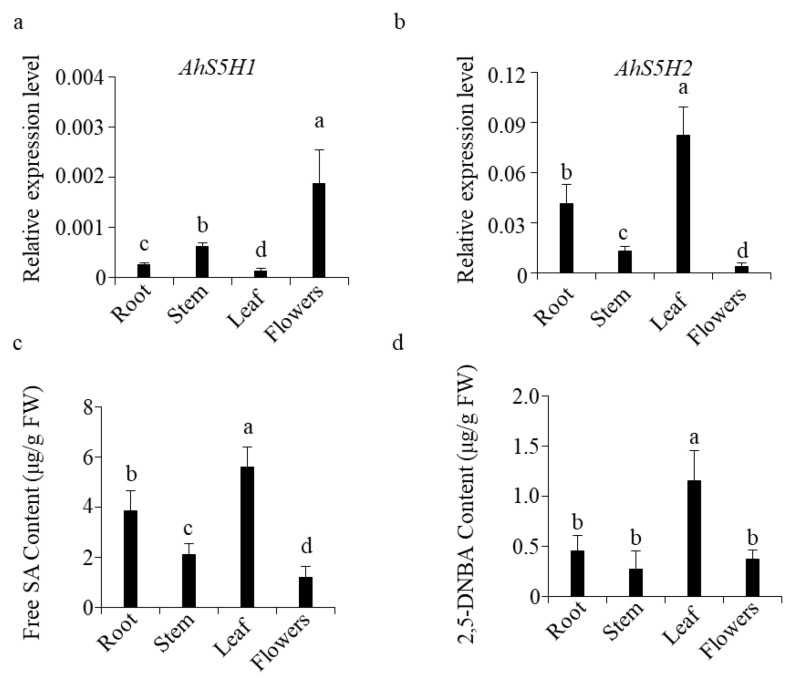
Expression of *AhS5Hs* and accumulation of SA and 2,5-DHBA. *AhS5H1* (**a**) and *AhS5H2* (**b**) expression in different tissues of peanut. Roots, leaves, stems and flowers were collected from plants grown in the paddy field at stage of flowering. Transcript level was determined by qRT-PCR using *AhACTIN* as the reference gene. The accumulation of SA (**c**) and 2,5-DHBA (**d**). Values are means ± SD (*n* = 3). Columns marked with different letters (a–d) indicate significant differences, as analyzed by the SPSS software version 22.0 (Duncan’s multiple range test, α = 0.05).

The expression levels of *AhS5H2* were highest in leaves, followed by roots, stems, and flowers (Figure 7b). Interestingly and surprisingly, the order of *AhS5H1* expression levels was completely reversed(Figure 7a), suggesting that the expression of *AhS5H1* and *AhS5H2* in peanut was tissue-specific.

The determination of phenolics showed that free SA content was highest in leaves among the tissues tested(Figure 7c), followed by roots, stems, and flowers. So was the level of 2,5-DHBA (Figure 7d). The relationships between the expression level of salicylate hydroxylase genes and SA content are shown in Table S2. *AhS5H2* expression levels were highly correlated with SA content (r = 0.9600) in roots, stems, leaves, and flowers. In contrast, *AhS5H1* was highly negatively correlated (r = −0.8038). It is possible that *AhS5H2* prefers to be expressed in tissues with high SA content in order to exert a stable function due to its higher *K_m_* value.

## 3. Discussion

The roles of SA during immune responses and senescence have been widely investigated in different plant species [35,36,37,38,39]. However, the biological importance of SA in peanut resistance remains deficient. Our results showed that the resistance of peanuts to many common pathogens could be induced by the SA. Therefore, the role of the SA-signaling pathway in peanut disease resistance deserves further investigation.

Phytochemical protection can easily cause environmental safety problems. In contrast, the acquisition of resistant materials seems more worthy of adaptation. The development of resistant varieties typically relies on the deployment of dominant resistance (*R*) genes, whose products mediate the recognition and protection against specific pathogen strains. However, resistance mediated by a single *R* gene frequently lacks durability, because pathogens can easily lose or mutate their *R* gene’s cognate molecule (effector) [12]. Resistance is not easily overcome by combining multiple *R* genes in a genotype. It implies that extra effort is needed to find more *R* genes [13,14]. On the contrary, it is more efficient to look for the so-called *S* genes that are conserved in various species of plants [40]. No *S* genes are yet known to confer resistance to pathogens in peanuts. In this work, we identified two *S* genes (*AhS5H1* and *AhS5H2*) with some similar characteristics to those previously described in rice and Arabidopsis [25,26,27,30] (Figure 2). Both of the proteins (AhS5H1 and AhS5H2) converted SA to 2,5-DHBA in vitro (Figure 3), but also showing differences in the kinetic properties. For example, both OsS5H-1 and OsS5H-2 in rice are inhibited by the substrate (SA), showing very low *K_si_* values (9.10 and 1.60 μM) [41]. In contrast, AhS5H1 and AhS5H2 were not inhibited by SA, showing a hyperbolic response when SA levels were increased (Figure 3d). This result suggests that the role of rice enzymes in hydroxylating SA is probably more significant at low SA concentrations, while the peanut enzymes may have a major role at higher SA concentrations.

To further analyze the roles of *AhS5H1* and *AhS5H2*, we generated overexpressing lines in Columbia-0 (Col-0) background Arabidopsis. In this study, overexpression of *AhS5H1* or *AhS5H2* decreased SA content, suggesting that both enzymes could have a similar role in planta. In the meantime, the content of 2,5-DHBA was significantly increased in the corresponding overexpression lines. The results of the phenolic content assay were consistent with those of the *AtDMR6* overexpression lines in previous reports [24,25]. Therefore, the functions of AHS5H1 and AHS5H2 were highly similar to those of AtDMR6.

The inactivation of orthologs of *AtDMR6* caused an increase in the SA content in plants. It always confers resistance to more than one pathogen species or to most races or strains of the same pathogen [28,31,42]. In this study, SA treatment was used to simulate the increase in endogenous SA in peanut, and our results indicated that peanut resistance to multiple pathogens was induced by exogenous SA. The phenotype of increased resistance was similar to that of those mutants. Moreover, *Ah5H1* and *AhS5H2* overexpression lines displayed low SA levels and were more susceptible to *Pst* DC3000. This also proves that the candidate S genes, *AhS5H1* and *AhS5H2*, are important for their effect on disease resistance.

Systemic acquired resistance (SAR) is a defense reaction that can be aroused when plants are infected by pathogens. It is effective against bacterial, fungal and virus pathogens [43,44,45] through the concerted activation of pathogenesis-related (*PR*) genes [46,47]. Generally, pathogen-induced SAR relies on an activated SA-dependent pathway [43], while *PR10* encodes SA-inducible *PR* [48]. Additionally, none expressor of pathogenesis-related genes 1 (*NPR1*) has been identified as an important component for the SA-regulated resistance [49]. Both *AhNPR1* and *AhPR10* are associated with *Phomopsis liquidambaris* B3-induced SA-dependent signaling in suppressing root rot [11]. Our results confirmed that the levels of *AhNPR1* and *AhPR10* expression were significantly induced after being treated with chitin. Simultaneously, the *Ah5H1* and *AhS5H2* were remarkably up regulated. In Arabidopsis, the SAR against pathogens has been associated with the accumulation of salicylic acid (SA) and the expression of the pathogenesis-related proteins AtPR1, and AtPR2 [38]. Overexpression of each *AhS5H* in Arabidopsis abolished the induction of *AtPR1* or *AtPR2* upon chitin treatment (Figure 6e,f). Our study demonstrated that expression of *AhS5H1* and *AhS5H2* was affected during defense responses.

AhS5H1 and AhS5H2 were highly similar in biochemical function and response to chitin treatment. However, the transcriptional levels of *AhS5H1* and *AhS5H2* were tissue-specific. Compared with AhS5H1, AhS5H2 had a higher *K_m_* value. This means that a higher content of SA is required to achieve an optimal enzymatic reaction rate for AhS5H2. The detection of compounds also attested that the expression of *AhS5H2* was higher in tissues containing more adequate SA. 

In conclusion, the results presented here demonstrate that peanut plants have two salycilic hydroxylase enzymes. *AhS5H1* and *AhS5H2* were identified as candidate susceptibility genes in peanuts for the first time. Both hydroxylases have similar biochemical functions. Meanwhile, the expression of *AhS5H1* and *AhS5H2* was defense-associated and tissue-specific in peanut.

## 4. Materials and Methods

### 4.1. Plant Materials and Treatments

The peanut variety Baisha1016 (*Arachis hypogaea* cv. Baisha1016, as a hyper-susceptible variant to all the pathogens involved in this study) was used for all inoculations. At 28 °C in darkness, pre-germinated seeds were sown in a controlled climate room that was maintained at 28 °C, with a 12 h photo-phase. The Arabidopsis plants were grown on potting soil at 21 °C with 16 h of light and 75% relative humidity. The Arabidopsis and peanut seedings were treated with 5 mM MES (4-morpholine ethanesulfonic acid, pH 5.8) buffer containing 1 mM SA or 100 μg/mL chitin, and the same volume of DMSO solvent was used as the control.

### 4.2. Phylogenetic Analysis

The molecular weights were deduced using Lasergene ediseq ver.7.1 software. Homologs of DMR6 and DLO proteins were retrieved from GenBank databases using protein blast (available online: https://blast.ncbi.nlm.nih.gov/Blast.cgi, accessed on 18 January 2022). The multiple sequence alignment was carried out via DNAMAN6.0, and a phylogenetic dendrogram was generated with MEGA 7.0 using the neighbor-joining method.

### 4.3. Pathogen Inoculation

After 24 h of SA treatment, peanuts were inoculated with the pathogens, and untreated plants served as controls. Three-week-old peanut plants were inoculated with a virulent *P. arachidicola* LN1 strain by spraying the spore suspension (2 × 10^6^ conidia/mL containing 0.1% Tween 20) as described by [50]. Disease severity was evaluated by PCR amplification of the relative biomass. Quantification was performed with primers from *P. arachidicola* 26S rDNA and the *AhACTIN* gene. Template DNAs from the inoculated leaves were collected 7 days after the inoculation. 

To evaluate resistance against Early Leaf Spot, three-week-old peanut plants were inoculated with *C. arachidicola* SY7 by foliar spraying (5 × 10^5^ conidia/mL spores containing 0.005% Silwet L-77), in which the *C. arachidicola* SY7 strain was isolated from the naturally infected peanut leaves. The number of disease spots was counted as described previously by Zanão Júnior et al. [51].

Baisha1016 susceptible to *E. arachidis* served as a host. Mycelium suspension was sprayed onto the one-month-old peanut leaves, and then incubated in a chamber under constant light conditions at 25 °C for lesion formation [52]. Disease severity was also evaluated by PCR amplification of the relative biomass. Quantification was performed with primers from *E. arachidis* 26S rDNA and the *AhACTIN* gene. The inoculated leaves were collected 10 days after the inoculation.

Subsequently, four-week-old peanut plants were inoculated with *Sclerotium rolfsii* Sacc CH isolate grown on PDA with slices of filter paper (about 10 mm × 4 mm) on the agar for five days. The filter paper, grown over by CH, was pinned around the bottoms of peanut. The lengths of the disease lesion were measured at 6 dpi (days post inoculation).

The Arabidopsis inoculation protocol was performed as described by Zeilmaker et al. [25]. To measure the growth of *P. syringae pv tomato* DC3000, Arabidopsis plants at the age of three weeks were subjected to a bacterial suspension (optical density 0.05) supplemented with 0.02% silwet L-77. Leaf samples (four plants per line; three leaves per plant) were collected for enumeration of colonies at 0 and 3 days after inoculation.

### 4.4. Protein Expression and Enzyme Assays

The sequences of *AhS5H1* and *AhS5H2* were amplified from cDNAs of *Arachis hypogaea* L. leaves. Both were cloned into a modified pGEX-tag vector with 3 × Myc at the C-terminus of the recombinant protein [53]. After transformation of each plasmid into *Escherichia coli* BL21 (DE3), protein expression was induced by the addition of 0.2 mM IPTG grown at 28 °C for 6 h. Recombinant proteins were purified using Glutathione Sepharose 4B (GE Healthcare, Chicago, IL, USA).

The enzymatic activity assay was performed as described previously [30]. Briefly, a total volume of 100 μL reaction mixture contains 5 μg recombinant protein in the reaction buffer (1 mM 2-oxoglutaric acid, 1 mM sodium ascorbate, 0.4 mM FeSO_4_, 0.1 mg/mL catalase, 5 mM DTT, 50 mM phosphate buffer at pH 6.8) with different concentrations of SA. The reaction was incubated at 40 °C for 30 min and stopped by adding two volumes of acetonitrile and boiling for 1 min. The supernatant was analyzed by HPLC.

### 4.5. Determination of Metabolites

The leaves and other tissues of the peanut from wild-type and transgenic Arabidopsis plants were collected and stored at −80 °C. The samples were extracted with 90% aqueous methanol containing 0.1% formic acid as described previously [54].

Chemicals were separated by a DIKMA (Beijing, China)C18 column (250 × 4.6 mm, 5 μm) on a Waters 2695 separation module (Waters, Shanghai, China). The elution conditions were at a flow rate of 1 mL/min with a gradient program of 12% acetonitrile for 15 min up to 75% in 8 min, then to 95% in 1 min, then the column was washed and equilibrated to the initial conditions. The absorption at 230 nm was detected and concentrations were calculated by the peak area of samples according to a standard curve.

### 4.6. Reverse Transcribed Quantitative (qRT-PCR) Analysis

After removing possible DNA contamination, two micrograms of total RNA were reverse transcribed with random hexamers and oligo(dT)18 primers using M-MLV reverse transcriptase (Takara, Kusatsu-shi, Japan). The relative transcript levels were quantified using SYBR Green PCR Master Mix (Takara) and normalized to *AhACTIN* or *AtACTIN2*. The relative expression level of each gene was analyzed using the delta-delta C_t_ method. Gene-specific primers used in qRT-PCR are listed in Supplementary Table S1.

## 5. Conclusions

Two salicylate hydroxylase genes were successfully predicted and identified within the peanut genome, marking a significant milestone as the inaugural discovery of susceptible genes in this particular crop. The salicylate hydroxylase exhibited analogous biochemical functions, thereby resulting in a reduction of the SA content within transgenic Arabidopsis. However, their expression exhibited tissue specificity.

## Data Availability

The data presented in this study are available upon request from B.L.

## Supplementary Materials

The supporting information can be downloaded at https://www.mdpi.com/article/10.3390/ijms241814210/s1.
