# Supplementary material for: Overexpression of the First Peanut-Susceptible Gene, AhS5H1 or AhS5H2, Enhanced Susceptibility to Pst DC3000 in Arabidopsis
Source: Int J Mol Sci. 2023 Sep 18;24(18):14210. doi: 10.3390/ijms241814210 (PMC10531710; doi:10.3390/ijms241814210)
Supplement: Supplementary file 1 [file ijms-24-14210-s001.zip › Supplementary Material 2.pdf]

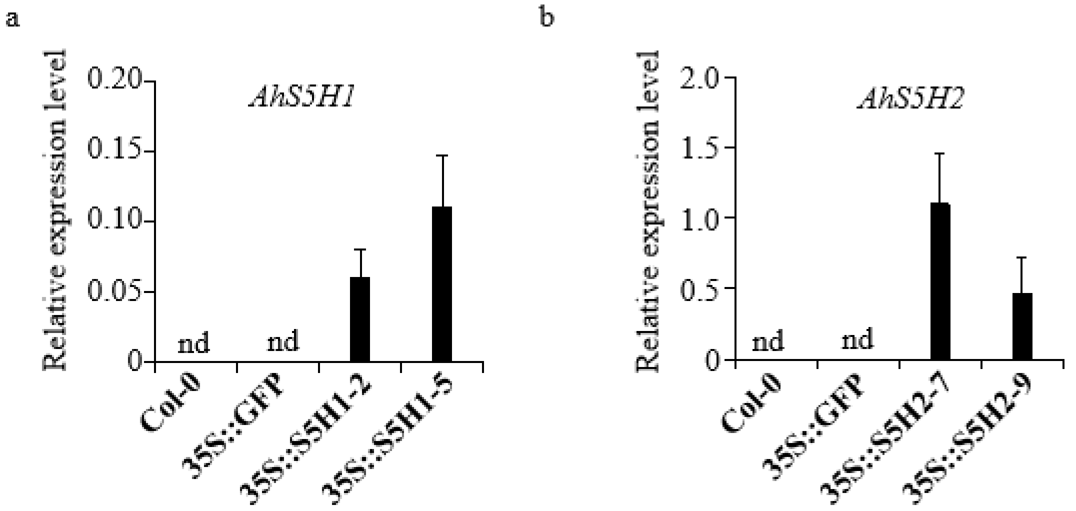

Figure S1. Expression of *AhS5H1* and *AhS5H2* in transgenic Arabidopsis. Expression level of *AhS5H1* (a) and *AhS5H2* (b) in transgenic Arabidopsis plants. Expression was determined by qRT-PCR using *AtACTIN2* as the reference gene. Values are means ± SD (n = 3). Nd, not detectable.

Table S2. The correlation between compounds content and *AhS5H* transcription level (r value).

Compounds	*AhS5H1*	*AhS5H2*
SA	-0.8438	0.9600
2,5-DHBA	-0.7682	0.9319

Note: ."+" indicates positive correlation; "-" indicates negative correlation. 0.8-1.0 indicates extremely high correlation; 0.6-0.8 indicates high correlation; 0.4-0.6 indicates moderately correlated; 0.2-0.4 indicates weak correlation; 0.0-0.2 indicates very weak or no correlation. The correlations between compounds content and *AhS5H* transcription levels were analyzed using the Pearson method of Microsoft Excel.
